# Health Literacy, Computer Skills and Quality of Patient-Physician Communication in Chinese Patients with Cataract

**DOI:** 10.1371/journal.pone.0107615

**Published:** 2014-09-16

**Authors:** Xianchai Lin, Mei Wang, Yajing Zuo, Mingge Li, Xiaofeng Lin, Siping Zhu, Yongxin Zheng, Minbin Yu, Ecosse L. Lamoureux

**Affiliations:** 1 State Key Laboratory of Ophthalmology, Zhongshan Ophthalmic Center, Sun Yat-sen University, Guangzhou, China; 2 Department of Ophthalmology, Sun Yat-sen Memorial Hospital, Sun Yat-sen University, Guangzhou, China; 3 Shantou Central Hospital, Shantou, China; 4 Singapore Eye Research Institute, Singapore National Eye Centre, Singapore, Singapore; 5 Duke-National University of Singapore Graduate Medical School, Singapore, Singapore; 6 Centre for Eye Research Australia, University of Melbourne, Melbourne, Australia; Medical University Graz, Austria

## Abstract

**Purpose:**

The aim of the study was to assess levels of health literacy and computer skills in Chinese patients with cataract, and their impact on the doctor-patient relationship.

**Methods:**

We undertook a cross-sectional study of cataract patients scheduled for cataract extraction procedures in Guangdong Province, China. Generic health literacy was assessed using 3 established screening questions. Adequate computer skills was determined if patients had used a computer and routinely used search engines on the Internet. Socio-demographic measures (e.g., age, sex, education) were obtained from a standardized interview. Participants who indicated that they could not understand what their doctors mean were considered to have had poor patient-physician communications.

**Results:**

Of the 211 participants, 92 (43.6%) had inadequate health literacy and 204 (96.7%) inadequate computer skills. In multivariate analysis, females were more likely to have inadequate health literacy (odds ratio = 2.5, 95% confidence intervals [CI]: 1.3 to 4.7). People with inadequately health literacy were more likely to have a poor patient-physician communication (odds ratio = 3.5, 95% CIs: 1.3 to 9.0). Similar associations were found for inadequate computer skills.

**Conclusion:**

Chinese elderly patients with cataract have inadequate health literacy and very limited computer skills, which place them at high risk of misunderstanding and mismanaging their ocular conditions. Patient education information other than online materials may improve the eye care and outcomes of these patients.

## Introduction

According to the Institute of Medicine, health literacy is defined as “the degree to which individuals can obtain, process and understand basic health information and services needed to make appropriate health decisions”. [1] Health literacy goes beyond the ability to read or write (general literacy), and is more useful to reflect patient’s health care seeking ability than general literacy. Nearly 91% of adults in China and 25% of adults in the USA lack the skills to fully comprehend verbal and written information in a healthcare setting. [2], [3] Inadequate health literacy not only compromises health care, it also accounts for up to 5% of health care costs annually according to a recent systematic review [4].

Cataract is the leading cause of blindness and visual impairment worldwide, and cataract surgery is considered as one of the most cost-effective interventions in healthcare. [5] According to the American Academy of Ophthalmology, cataract surgery is indicated when the cataract causes decreased visual functioning to a level that interferes with patients’ activities of daily living or impacts on driving, work and other activities. [5] Given that it is an elective surgery, patients are supposed to take part in the surgical decision making and have a full understanding of potential benefits and risks. In addition, patients are frequently operated in an outpatient setting and much care and post-operative management is performed by individual patients outside of the hospital. Therefore, an adequate level of health literacy is critical in ensuring desirable surgical outcomes and proficiency in self-management, whereas poor health literacy may undermine their health care.

There is limited information about the levels of health literacy among patients with cataract. Furthermore, given that there is no or negligible effect of passive dissemination of printed education materials for health promotion, [6] there is an increasing interest in the availability of online information, but it is unclear how many patients in China are indeed equipped with adequate computer skills. Therefore, the objective of this study was to evaluate the levels of health literacy and computer skills among patients with cataract, and the influences of limited health literacy and computer skills on patient-physician communication in a Chinese population with cataract.

## Methods

### Study Population

All patients awaiting a cataract extraction procedure in one or both eyes at the Second Affiliated Hospital, Sun Yat-sen University, Guangzhou, Southern China, comprised the participants of this study. Inclusion criteria were age 18 years or older and no severe cognitive impairment. The study adhered to the Declaration of Helsinki, and it was a voluntary and anonymous survey. The waiver of written consent and the overall study was approved by the Zhongshan Ophthalmic Center Institutional Review Board.

### Health Literacy and Computer Skills

An interviewer-administered questionnaire was used to collect information on health literacy, computer skills, and socio-demographic variables. Health literacy was assessed by asking 3 standardized screening questions [7]: (1) How often do you have someone help you read hospital materials? (2) How often do you have problems learning about your medical condition because of difficulty reading hospital materials? and (3) How confident are you filling out medical forms by yourself? Each question was scored on a 5-point scale in which a higher score indicates lower literacy. The participants were told that these questions were designed to assess their health literacy instead of vision problems. As such, those who indicated that difficulty in reading hospital materials was solely due to a visual condition were categorized as health literate. Based on previous literature [8], [9], scores were summed a priori so that a summary score greater than 10 was classified as low health literacy and a score of 10 or lower as adequate health literacy.

Computer skills were assessed by asking 3 questions: (1) Have you ever used a computer? (2) Can you turn on a computer and browse the internet? and (3) Do you use search engines on the internet? Only those who answered “yes” to all the questions were considered to have adequate computer skills. Otherwise, participants were categorized as having limited computer skills.

Other collected information included age, sex and education (polytechnic/University; secondary education; primary education; no formal education). Quality of patient-physician communication was assessed by asking 1 question: Did any of the health care provider(s) explain whether or not to have cataract surgery in a way that you could understand? Those who replied “I cannot understand” were considered to have poor patient-physician communication.

### Eye Examination

Routine clinical assessments were performed. [10] VA was measured using a Snellen visual acuity chart at a distance of 6 meters. If the patient cannot read the largest letter at 6 meters, he or she would be moved closer one meter at a time. If no letter was identified at 1 meter, VA was assessed as *Counting Fingers, Hand Movements, Perception of Light,* or *No Perception of Light*. Both present VA (PVA) and best corrected VA (BCVA) were obtained. Bilateral visual impairment was defined as VA worse than 20/40 in the better-seeing eye.

### Statistical Analysis

Logistic regression was used to evaluate the odds ratio (OR) and the 95% confidence interval (CI) for the association between potential factors (e.g., age, sex and education) and limited health literacy and computer skills. Variables identified as significant (p<0.05) were retained in multivariate models. Statistical analyses were performed using the software STATA (Version 8.2, Stata Corp., College Station, TX).

## Results

Among 215 patients surveyed, 211 responded (98% response rate). The sample average age was 69.5 yr. (standard deviation: 11.4) and 53.6% were female. **Table 1** shows the baseline the participants’ characteristics. A total of 92 (43.6%) had inadequate healthy literacy and 204 (96.7%) had inadequate computer skills. People with inadequate health literacy were more likely to be female, factory workers and home makers than those with adequate health literacy.

**Table 1:** Sociodemographic and clinical characteristics of the participants with cataract.

	Persons withinadequatehealth literacy	Persons withadequatehealth literacy	Persons withInadequatecomputer skills	Persons withadequatecomputer skills
All	92 (43.6)	119 (56.4)	204 (96.7)	7 (3.3)
Age groups				
18–39 years	1 (1.0)	4 (3.4)	5 (2.4)	0 (0.0)
40–59 years	11 (12.0)	13 (10.9)	23 (11.3)	1 (14.3)
60+years	80 (87.0)	102 (85.7)	176 (86.3)	6 (85.7)
Gender (female)	61 (66.3)	52 (43.7)	109 (53.4)	4 (57.1)
Education level				
Secondary education or higher	28 (34.2)	60 (52.6)	85 (44.5)	3 (60.0)
No formal or primary education	54 (65.8)	54 (47.4)	106 (55.5)	2 (40.0)
Occupation				
Service work	11 (12.0)	24 (20.2)	33 (16.2)	2 (28.6)
Professional/officer	7 (7.6)	13 (10.9)	18 (8.8)	2 (28.6)
Factory work/home making	74 (80.4)	82 (68.9)	153 (75.0)	3 (42.8)
Income level				
>RMB2000	12 (13.0)	24 (20.2)	34 (16.7)	2 (28.6)
≤RMB2000	80 (87.0)	95 (79.8)	170 (83.3)	5 (71.4)

Data presented are means (standard deviations) or number (%), as appropriate for variable.

**Table 2** shows the associations of socio-demographic variables with health literacy and computer skills. Females and those with no education or primary education level were more likely to have inadequate health literacy. In multivariate analysis, being female remained significantly associated with inadequate health literacy (OR [odds ratio] = 2.5; 95% CI [confidence intervals]: 1.3 to 4.7) compared with males, after controlling for age, educational level, income and occupation.

**Table 2:** Associations of socio-demographic variables with health literacy and computer skills.

	OR (95% CI) for the presence of inadequatehealth literacy		OR (95% CI) for the presence of inadequatecomputer skills	
	Univariate analysis	Multivariate analysis*	Univariate analysis	Multivariate analysis*
Age	1.2 (0.9 to 1.6)	1.2 (0.8 to 1.5)	0.9 (0.5 to 2.0)	0.8 (0.4 to 1.8)
Gender (female)	**2.5 (1.4** to **4.5)**	**2.5 (1.3** to **4.7)**	1.2 (0.3 to 5.3)	0.8 (0.1 to 7.1)
Education level				
Secondary education or higher	Reference	Reference	Reference	Reference
No formal or primary education	**2.1 (1.2 to 3.9)**	1.7 (0.8 to 3.3)	0.5 (0.1 to 3.3)	0.9 (0.1 to 8.8)
Occupation				
Service work	Reference	Reference	Reference	Reference
Professional/officer	1.2 (0.4 to 3.8)	0.8 (0.2 to 3.1)	1.8 (0.2 to 14.1)	1.5 (0.2 to 43.7)
Factory work/home making	2.0 (0.9 to 4.3)	1.0 (0.4 to 2.6)	0.3 (0.1 to 2.0)	0.6 (0.1 to 8.4)
Income level				
>RMB2000	Reference	Reference	Reference	Reference
≤RMB2000	1.7 (0.8 to 3.6)	1.4 (0.6 to 3.4)	0.5 (0.1 to 2.7)	1.3 (0.1 to 54.4)

OR = odds ratio; 95% CI = 95% confidence interval.

*Multivariate analysis adjusting for age, gender, educational level, income and occupation.

**Table 3** shows the associations of inadequate health literacy with poor patient-physician communication. In multivariate analysis adjusting for age, gender, education, income and occupation, inadequate health literacy (OR = 3.5, 95% CI: 1.3 to 9.0) remained significantly associated with poor patient-physician communication. A similar result was seen for the association of inadequate computer skills with poor patient-physician communication (OR = 12.7, 95% CI: 1.1 to 146.3) (**Table 4**).

**Table 3:** Association of inadequate health literacy with patient-physician communication.

	OR (95% CI) for poor patient-physician communication*	
	Univariate analysis	Multivariate analysis**
Inadequate health literacy	**3.6 (1.6 to 8.1)**	**3.5 (1.3 to 9.0)**
Education level		
Secondary education or higher	Reference	Reference
No formal or primary education	0.9 (0.4 to 2.1)	1.0 (0.4 to 3.1)
Income level		
>RMB2000	Reference	Reference
≤RMB2000	1.1 (0.4 to 3.1)	0.7 (0.2 to 2.6)
Occupation		
Service work	Reference	Reference
Professional/officer	0.5 (0.1 to 1.9)	0.1 (0.1 to 1.3)
Factory work/home making	0.5 (0.2 to 1.2)	0.3 (0.1 to 0.9)

OR = odds ratio; 95% CI = 95% confidence interval.

*Participants who indicated that they could not understand what their doctors mean were considered to have had poor patient-physician communications.

**Multivariate analysis adjusting for age, gender and socioeconomic measures (e.g., education, income and occupation).

**Table 4:** Associations of inadequate computer skills with patient-physician communication.

	OR (95% CI) for poor patient-physician communication	
	Univariate analysis	Multivariate analysis*
Inadequate computer skills	**20.4 (1.1 to 45.7)**	**12.7 (1.1 to 146.3)**
Education level		
Secondary education or higher	Reference	Reference
No formal or primary education	0.9 (0.4 to 2.1)	1.2 (0.4 to 3.6)
Income level		
>RMB2000	Reference	Reference
≤RMB2000	1.1 (0.4 to 3.1)	1.0 (0.3 to 3.7)
Occupation		
Service work	Reference	Reference
Professional/officer	0.5 (0.1 to 1.9)	0.1 (0.1 to 220.5)
Factory work/home making	0.5 (0.2 to 1.2)	0.4 (0.2 to 340.8)

OR = odds ratio; 95% CI = 95% confidence interval.

*Multivariate analysis adjusting for age, gender and socioeconomic measures (e.g., education, income and occupation).

## Discussion

In the Chinese patients with cataract, more than 43% had inadequate health literacy and approximately 96% had no computer skills for seeking online health information. The problem was especially prevalent among the females. In addition, there was a significant association between inadequate health literacy and poor patient-physician interaction, after controlling for the effect of socioeconomic measures such as education and income. These findings suggest that people with inadequate health literacy are at greater risk of patient-physician misunderstanding as well as miscommunicating their perceptions of risks and benefits of treatment options. The results highlight an important target for interventions to improve the quality of eye care for those with cataract. Our study has both health and social implications in China, in which strain on the doctor-patient relationship has continued to worsen and there is an increasing level of dissatisfaction among patients for their poor access to health care.

Our finding of a high prevalence of low health literacy among cataract patients is consistent with those reported at public hospitals in America, in which over one third of English-speaking patients had inadequate or marginal health literacy. [11] In addition, we found that 7.6% officers and professionals had low health literacy, suggesting that health literacy problems are not confined to those with high levels of education attainment. The most commonly used instruments, the Rapid Estimate of Adult Literacy in Medicine (REALM), Test of Functional Health Literacy Assessment (TOFHLA), Newest Vital Sign (NVS) and their derivatives, are only available in English and Spanish and they are not very culturally sensitive, precluding their use in the Chinese communities. [12]–[15] In addition, those measures are relatively time-consuming and can invoke feeling of shame. [9] By contrast, the 3 brief screening questions in this study have been widely validated against REALM, TOFHLA and other health literacy instruments, and can be easily administered in population screening and routine clinical settings. [7] While the current study did not use a detailed tool to validate these screening questions, it is valid to argue that there is no gold standard for measuring health literacy worldwide, especially for Mandarin-speaking Chinese populations. [16] More importantly, these questions have a value of their own: they provide a direct approach to investigate if patients are able to comprehend health materials than traditional health literacy assessment tools, and are useful for identifying patients at higher risk of misunderstanding or ignoring medical advice in a healthcare setting [9].

Health literacy is considered as patient’s currency for negotiating the health care system. [16] Previous studies have documented the association between low health literacy and poor eye health. For example, it has been reported previously that low health literacy is associated with the presence of diabetic retinopathy among those with type 2 diabetes. [17] Among patients with glaucoma, those with a lower level of health literacy are more likely to be have poor medication adherence and worsening of visual field. [18]–[21] There are multiple reasons that explain why health literacy has an impact on health, one of which being the misunderstanding and miscommunication between patients and physicians. To our knowledge, this is the first study to document the link between inadequate health literacy and poor patient-physician communication among patients who were scheduled for cataract surgery. Given that cataract surgery is an elective procedure, it has been assumed that patient should participate in a shared decision making process, in which the patient and the provider share information and determine when cataract surgery is needed. [22] While health practitioners expect patients to have more responsibility for self-care and shared decision, low health literacy may become a significant barrier to patient-centered care. This is heightened by the fact that China’s health system is increasingly fragmented and technically sophisticated. While culturally relevant interventions are needed to promote patient autonomy, there is a lack of financial incentives to influence physician practice patterns.

Given that some misunderstanding exists between patients and their providers, it is hoped that easily accessible health education brochures and pamphlets will help patients clarify their understanding and make informed decision. Also, online health education technologies such as social media, podcasts, apps, and interactive formats are increasing available. [23] Unfortunately, the finding that only 5% of patients had computer skills suggests that these cataract patients are not prepared for online self-education. There is therefore a need to develop and validate new methods for conveying medical information to patients with limited health literacy in urban China.

The inclusion of a relative large sample of patients with cataract is a strength of this study, but it has a few limitations as well. The cross-sectional design of this study limits our ability to document the true causal relationships between health literacy and health care experience, and many important confounders that would explain the relationship may have been unmeasured. The use of self-report instruments could not fully reflect the complex communication process between patients and providers and may have also introduced recall bias. The study employed convenience sampling, which may have introduced selection bias. In addition, our study sample reflects a local population and the statistical results are not generalizable to other populations, given the wide range of variation related to different populations, counties, cultures and socioeconomic levels. Finally, while the 3 health literacy items have been widely validated, the scale may be mingled with other constructs. For example, the third item is about confidence and reflects psychosocial or emotional aspects that may not be fully relevant to health literacy.

In summary, inadequate health literacy and limited computer skills are prevalent in patients with cataract in urban China. Our study highlights a need to validate a simpler and effective screening questionnaire for health literacy assessment that can be incorporate into medical examinations. Limited health literacy is associated with poor patient-physician communication. Further research is needed to determine whether interventions to address low health literacy can enhance doctor-patient communication.
